# The effectiveness of the biannual application of silver nitrate solution followed by sodium fluoride varnish in arresting early childhood caries in preschool children: study protocol for a randomised controlled trial

**DOI:** 10.1186/s13063-015-0960-2

**Published:** 2015-09-25

**Authors:** Chun-Hung Chu, Sherry Shiqian Gao, Samantha KY Li, May CM Wong, Edward CM Lo

**Affiliations:** Faculty of Dentistry, University of Hong Kong, 34 Hospital Road, Hong Kong, SAR China

**Keywords:** Silver nitrate, Fluoride varnish, Silver diamine fluoride, Caries

## Abstract

**Background:**

The application of 38 % silver diamine fluoride (SDF) has been shown to be effective in arresting early childhood caries (ECC). Since SDF is not available in certain countries, some dentists use adjunctive application of 25 % silver nitrate (AgNO_3_) and 5 % sodium fluoride (NaF) to arrest ECC. This randomised controlled trial will systematically compare the efficacy of a 25 % AgNO_3_ solution followed by 5 % NaF varnish with that of a 38 % SDF solution in arresting ECC when applied at half-yearly intervals over a 30-month period.

**Methods/Design:**

This study is a randomised, double-blinded, non-inferiority clinical trial. The hypothesis tested is that adjunctive application of 25 % AgNO_3_ followed by 5 % NaF is at least not appreciably worse than a 38 % SDF in arresting ECC. Approximately 3100 kindergarten children aged 3–4 years will be screened and at least 1070 children with caries will be recruited. This sample size is sufficient for an appropriate statistical analysis (power at 90 % (*β* = 0.10) with a 2-sided type-I error of *α* = 0.05), allowing for an overall 20 % drop-out rate. The children will be randomly allocated into 2 groups to treat their caries over a 30-month period:

- Group A – biannual adjunctive application of a 25 % AgNO_3_ solution and a 5 % NaF varnish, and
- Group B – biannual adjunctive application of a 38 % SDF solution followed by a placebo varnish.

Clinical examinations will be conducted at 6-month intervals. Primary outcome measured is the number of active caries surfaces which are arrested. Information on confounding factors such as oral hygiene habits will be collected through a parental questionnaire.

**Discussion:**

We expect that adjunctive application of 25 % AgNO_3_ solution and 5 % NaF varnish and of 38 % SDF solution can both effectively arrest ECC. Lower concentrations of silver and fluoride are contained in 25 % AgNO_3_ and 5 % NaF, respectively, than in 38 % SDF; therefore, AgNO_3_/NaF are more favourable for use in young children. Because its use for caries management is painless, simple, low-cost, and approved in many countries, AgNO_3_/NaF could be widely recommended and promoted as an alternative treatment to conventional invasive management of ECC.

**Trial registration:**

ClinicalTrials.gov: NCT02019160. Date of registration: 11 December 2013.

**Electronic supplementary material:**

The online version of this article (doi:10.1186/s13063-015-0960-2) contains supplementary material, which is available to authorized users.

## Background

### Early childhood caries

Early childhood caries (ECC) is defined as the presence of one or more decayed, missing (due to dental caries), or filled tooth surfaces in the primary teeth of a child of 71 months of age or under [1]. Despite advances in clinical care and dental research, ECC remains a challenging problem. The United States (US) Centers for Disease Control and Prevention has reported that 28 % of all US toddlers and preschoolers are affected by ECC and that nearly half of US children have ECC before entering kindergarten [2]. ECC cause pain and infection, and advanced caries will progress into the tooth pulp to eventually form a dental abscess [3]. If cases remain untreated they lead to tooth loss, which affects dentition. Poor dentition significantly affects children’s nutrition and consequently their growth, development, and general health. Socially disadvantaged children, such as those from poor families and those whose parents have low educational levels, are disproportionately affected [4, 5]. The FDI World Dental Federation *Oral Health Atlas* reported that millions of children are suffering from untreated ECC; however, with its great shortage of community dentists, the present dental care delivery system cannot cope with this high ECC prevalence [6]. Moreover, prevailing restorative methods for preventing and treating ECC are neither available nor affordable to children from disadvantaged families, who exhibit a particularly high caries risk [7]. Although there have been efforts to treat ECC in developing areas (e.g., using mobile dental clinics with portable equipment), in most cases the technology has proven to be too complicated for sustained use [8]. The cost of basic sets of instruments, dental materials and infection-control products is also too high. In this situation, a new approach for managing ECC is needed. This study uses a non-operative strategy for caries management in preschool children.

**Fig. 1:**
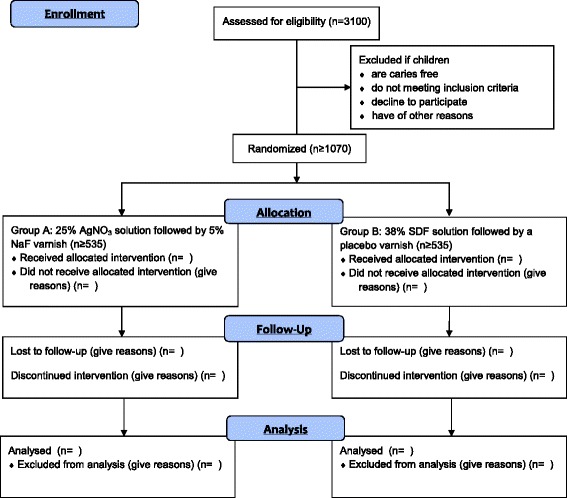
Flow diagram of the clinical trial

### Silver diamine fluoride

Fluoride was introduced to prevent caries more than 50 years ago [9]. Clinical studies have demonstrated that using 38 % silver diamine fluoride (SDF) can prevent and arrest ECC [10–15]. A literature review of SDF suggested that 38 % SDF can be an effective agent in preventing new caries and arresting dental caries in the primary teeth of children [16]. Another review concluded that SDF is an effective, efficient, and equitable caries-control agent [17]. Milgrom and Chi [18] advocated SDF therapy as an important prevention-centred caries management strategy during critical early childhood periods. The clinical trial by dos Santos et al. [19] concluded that 38 % SDF was better than interim restorative treatment with a glass ionomer for arresting ECC. Nevertheless, 38 % SDF exhibits a high fluoride concentration (44,800 ppm), which has concerned some researchers because of the possible onset of dental fluorosis [20]. Although SDF is used in countries in Asia and South America, SDF has not yet been approved for use in many European countries or in the USA [21].

### Adjunctive application of silver nitrate solution and sodium fluoride varnish

Although dental fluorosis can develop with increased fluoride dosage, fluoride use under professional instruction is generally safe and effective even in young children [9]. Sodium fluoride (NaF) varnish at 5 % contains 22,600 ppm of fluoride ions and is a common professionally applied topical agent in caries management. A literature review on the use of NaF varnish in managing ECC in children found that fluoride varnish appears to be the easier caries prevention method for both the dentist and the child [22]. However, NaF varnish is not effective in arresting dentine caries [10, 13]. Duffin [23] proposed using silver and fluoride ions from 25 % silver nitrate (AgNO_3_) solution followed by 5 % NaF varnish to arrest caries. A solution of 25 % AgNO_3_ and 5 % NaF varnish are accepted by most countries and their corresponding authorities such as the US Food and Drug Administration [23]. One laboratory study has suggested that 25 % AgNO_3_ solution and 5 % NaF varnish may be as effective as 38 % SDF in arresting dentine caries [24, 25]. However, a discussion of the use of AgNO_3_ and NaF by the Oregon Dental Association concluded that more research demonstrating efficacy and safety is required to meet the standard for evidence-based dentistry [21]. A literature search on PubMed on 1 December 2014, however, found that no research group has thus far published a well-designed study on the effectiveness of AgNO_3_ and NaF in the control of ECC.

### Objective

The overall objective of the randomised controlled trial is to systematically compare the effectiveness of a 25 % AgNO_3_ solution followed by 5 % NaF varnish with that of a 38 % SDF solution in arresting caries teeth among preschool children when applied at half-yearly intervals over a 30-month period.

### Hypothesis

In this non-inferiority randomised controlled trial, the hypothesis tested is that the biannual topical application of 25 % AgNO_3_ solution followed by 5 % NaF varnish is at least not appreciably worse than a 38 % SDF solution in terms of caries arrest in the primary teeth of preschool children.

## Methods/Design

### Trial design

This is a randomised, double-blind, non-inferiority clinical trial. The extension of the Consolidated Standards of Reporting Trials 2010 Statement will be followed [26]. The flowchart of this study is shown in Fig. 1.

### Setting

Kindergartens in Hong Kong who have joined our outreach dental service will be invited to join this study. Preschool children aged 3–4 years who have tooth decay and are attending the first year of kindergarten will be invited to join this study. An invitation letter will be sent to the parents explaining the purpose and procedures of this study. Written parental consent will be obtained before they are included in this clinical trial. We have trained an outreach team comprised of a dentist and a dental assistant to assess caries prevention and the caries-arresting effects of different treatments in preschool children in the kindergartens.

### Participants

The children included in the study should: a) be aged 3–4 years and are attending the first year of kindergarten, b) be generally healthy, c) have parental consent, and d) have at least 1 tooth with untreated caries with cavitation that extends into the dentine at baseline examination. Children who are uncooperative and difficult to manage, with severe forms of hypoplasia of fluorosis or other oral diseases, wearing orthodontic devices or under dental treatment, have major systemic diseases, or are on long-term medication will be excluded.

### Recruitment and screening

Oral health education will be provided to the children in the participating kindergartens, followed by a baseline oral examination. Potential participants will be recruited by trained field workers of the outreach team after the initial oral examination.

### Clinical examination

A clinical examination of the participating children will be performed in the kindergarten, primarily through a careful visual inspection with the aid of a disposable World Health Organisation Community Periodontal Index (CPI) probe (405/WHO probe, Otto Leibinger, Mühlheim, Germany) and a front-surface dental mirror with light-emitting diode intra-oral illumination (MirrorLite, Kudos Crown Limited, Hong Kong, China). The oral hygiene status will be measured using the visible plaque index (VPI). The buccal and lingual surfaces of 6 index teeth (55, 51, 63, 71, 75, and 83) will be examined. The presence or absence of visible plaque on the caries surface will also be recorded.

The tooth status (decayed, missing, filled surfaces (dmfs) score), tooth discolouration, and hyper-mobility will be recorded. Teeth with a caries lesion extending into the pulp or signs suggesting that the teeth are non-vital, such as tooth discolouration, hyper-mobility or abscess, will not be included in this study.

The caries will be diagnosed at the cavitation level. The carious lesion will be gently explored with the CPI probe in the centre of the lesion. Great care will be taken to avoid tooth damage during the probing. A lesion will be recorded as active if softness is detected upon gentle probing. If the dentine surface is hard to probing, it will be classified as an arrested caries [11–13, 15, 27]. All surfaces (i.e., buccal, lingual, mesial, distal, and occlusal for posterior teeth) of each tooth will be assessed. Active caries at the baseline that become arrested in the follow-up examinations will be used as the main treatment outcome.

A designated experienced outreach dentist will be the examiner throughout the 30-month study. The intra-examiner agreement on the plaque and caries assessment will be monitored and conducted in 10 % of the children at each stage of the study.

### Questionnaire survey

A validated parental questionnaire [4, 5] regarding their children’s oral hygiene habits (e.g., tooth-brushing and flossing), parent-assisted tooth-brushing, fluoride agent use (e.g., fluoride toothpaste and fluoride mouthwash), when brushing with fluoride toothpaste commenced, fluoride concentration in the used toothpaste, dental visit behaviour, snacking habits, parental educational level, family total income, and family status (single-parent or 2-parent households) will be administered at baseline and again at the 18-month and 30-month follow-up visits (Additional file 1). The questionnaires will also assess parental satisfaction with their child’s oral health and dental aesthetics. In the follow-up questionnaires, questions will also be asked about the potential post-treatment complications of the SDF application, such as pain in the treated tooth and gingival irritation around the treated tooth.

### Randomisation and treatment allocation

#### Allocation concealment

The participating children with caries will first be categorised as either: (1) having a high caries rate (or severe early childhood caries, as defined by the American Academy of Paediatric Dentistry [1]), which is defined as the presence of more than 3 untreated caries surfaces, or (2) having a low caries rate. The children will then be allocated by a stratified randomisation method using a computer-generated random-number table into the following 2 groups:

- Group A – biannual topical application of 25 % AgNO_3_ solution followed by 5 % NaF varnish; and
- Group B – biannual topical application of 38 % SDF solution followed by a placebo varnish.

#### Blinding

In this double-blind randomised clinical trial, the group allocation will be conducted by a dental assistant while the examiner will not know the allocation information. The examiner will not be informed of the treatment group allocation of the children and the children will not know the solution or varnish they receive throughout the study. The solution and varnish will be applied after the oral examination by another operator.

### Interventions

An independent operator will use a micro-brush to apply the appropriate solution and varnish according to the assigned treatment group after the oral examination. The Group A children will receive a 25 % AgNO_3_ solution (25 % silver nitrate, Gordon Labs, Carson, CA, USA) followed by a 5 % NaF varnish (Duraphat Varnish, Colgate-Palmolive, Endicott, NY, USA). The Group B children will receive a 38 % SDF solution (Saforide, Toyo Seiyaku Kasei Co., Osaka, Japan) followed by a placebo petroleum jelly varnish (Vaseline, Unilever, Englewood Cliffs, NJ, USA). The children in both groups will be instructed not to eat or drink for an hour after the application.

There is no adverse reaction of these two treatments, except the caries surfaces will become hard and black if the treatment has successfully arrested the caries lesions.

After the baseline examination, a report on the child’s oral health status will be provided to the parents with a note asking them to report to the study dentist if there are any side/adverse effects on the treated tooth and surrounding gum after treatment. During the follow-up treatments, the dentist will also look for related side/adverse effects, including blackening of the arrested caries surface, discoloured tooth, tooth hyper-mobility, and abscess formation.

### Follow-up evaluation

The follow-up oral examinations will be conducted every 6 months in the kindergarteners for 30 months by the same examiner using the same equipment, procedure, and diagnostic criteria as those used in the baseline examination. The oral hygiene and caries status of the child will be recorded. In addition, the presence of visible plaque and status of the included caries surfaces will be assessed. The examiner will not be the operator for the fluoride application.

### Outcome measure

The outcome measure (primary endpoint) is the number of soft (active) caries surfaces that become arrested (hardened) after 30 months. The outcome measure will be administered at baseline and at the 6-monthly follow-up examinations. Children and their parents have the right to receive dental treatment by other dentists. To our experience from previous clinical trials [11, 12], almost all participants will not be cared by other dental personnel, and we will include questions about the receipt of other dental treatment in baseline and follow-up interviews.

### Sample size and power calculation

This non-inferiority trial aims to measure the anti-caries efficacy of a 25 % AgNO_3_ solution followed by 5 % NaF varnish versus a 38 % SDF solution with a non-inferiority margin of −0.5 for the difference in mean number of arrested caries surfaces, which is considered clinically negligible (corresponding to an effect size = 0.25, assuming a standard deviation of 2.5 and true difference of 0) [11]. The estimated sample size, calculated with the help of a statistician, is based on the lower limit of the 2-sided 95 % confidence interval for the difference being set above the non-inferiority margin (−0.5), and statistical power of the study being set at 90 % (*β* = 0.10). The total required number of preschool children with caries will be 856 calculated with the use of statistical power analyses software, G*Power (version 3.1.7; Franz Faul, Universität Kiel, Kiel, Germany). Anticipating the caries prevalence to be 35 % [28] and the overall 30-month drop-out rate to be 20 % [11], the initial sample size for screening should thus be 3058 with a total of 1070 kindergarten children being recruited in the 2 study groups at the baseline examination.

### Statistical methods

A data management and statistical analysis protocol has been developed for the clinical trial. This details the procedures for data entry, management and cleaning, and data analysis.

The collected data will be entered into an Excel file (Microsoft Corp., Redmond, WA, USA) by two persons, and the data will be compared to minimise data entry errors. The intra-examiner agreement in caries diagnoses will be assessed by Cohen’s Kappa statistics. The level of statistical significance for all 2-sided tests will be set at 0.05. For the 1-sided tests, the level of statistical significance will be set at 0.025.

An intention-to-treat analysis will be performed in this study. We expect that the drop-out rates of the two groups will be similar because the treatment protocols and treatment outcomes (arrested caries with blackening of the lesion) are the same. If the number of children who strayed from the protocol (for instance, by not adhering to the prescribed 6-month intervention, or by being withdrawn from active treatment) are different between the 2 groups, a per-protocol analysis will be performed. In that case, only the patients who complete the entire clinical trial according to the protocol will be counted towards the final results.

#### Subject-level analysis

Although the data for the primary outcome itself may not be normally distributed [11], making use of the central limit theorem with the relatively large sample size that will be recruited in this research, the distribution of the mean would be normal. Non-inferiority of the 25 % AgNO_3_ solution followed by 5 % NaF varnish to 38 % SDF solution can be claimed if the lower limit of the confidence interval for the difference in the mean number of arrested surfaces is greater than the non-inferiority margin of −0.5. This test for non-inferiority will only be performed for the primary endpoint, the number of arrested surfaces after 30 months. Furthermore, a Student’s *T* test will be performed to study the effects of the treatment on the number of arrested caries surfaces at the 18-month examination. This research will also study the effect modification. The variables that are considered to possibly modify the treatment effects on the outcome variable include the patient’s gender, baseline caries experiences, treatment group assignment, oral hygiene habits, fluoride agent use, parent-assisted tooth-brushing, dental visit behaviour, snacking habits, parental educational level, total family income, and family status (single-parent or two-parent households).

Besides the primary outcome, Student’s *T* tests will be also used to test the between-group differences regarding the number of new caries surfaces, increment in the number of non-vital teeth, and number of hyper-mobile teeth across the follow-up examinations.

#### Surface-level analysis

To compare the differences in the time to caries arrest between the two treatment groups at the tooth surface level, a survival analysis will be adopted for the interval-censored data (because the time to arrest cannot be observed exactly but falls in the interval between the two examinations); this analysis will account for the possible correlation (clustering) between the observations of multiple surfaces from the same child. The statistical software SAS 9.2 for Windows (SAS Institute Inc., Cary, NC, USA) will be used for the analyses above.

### Ethical considerations

Ethics approval was obtained from the Institutional Review Board of the University of Hong Kong/Hospital Authority Hong Kong West Cluster (HKU/HAHKW IRB) (IRB reference number: UW 13–569). Informed consent will be sought from parents of each participating child prior to participation in the trial. In general, the trial will pose minimal risks to the participating children. In order to minimise risk, careful training has been provided to the field workers. A monitoring system is in place for potential adverse and serious adverse events, with a protocol for the management of these events and reporting at appropriate times to the HKU/HAHKW IRB.

All data from the baseline and follow-up examinations will be reviewed on a continuous basis by unblinded members (CHC and ECML) of the team to check on quality. The trial statisticians (SKYL and MCMW) will be asked to review the data at key points during the life of the trial. Reports will be submitted to the HKU/HAHKW IRB every 6 months for external quality control. All serious adverse events will be reported to the HKU/HAHKW IRB within 48 hours of being reported to the trial team.

## Discussion

This study is a non-inferiority clinical trial aiming to investigate whether biannual topical application of 25 % AgNO_3_ solution followed by 5 % NaF varnish is at least not appreciably worse than 38 % SDF solution in arresting caries in the primary teeth of children. The estimated sample size is based, with the help of a statistician, on the lower limit of the 2-sided 95 % confidence interval for the difference being set above the non-inferiority margin (−0.5) and statistical power of the study being set at 90 % (*β* = 0.10). As no other similar non-inferiority clinical trial has been conducted in this area, the non-inferiority margin of this trial cannot be inferred from previous studies. Since the number of dental caries surfaces is the integer and the hypothesis of this study is that 25 % AgNO_3_ solution followed by 5 % NaF varnish is at least not appreciably worse than 38 % SDF solution in arresting caries, the difference between the results (the number of caries arrested surfaces) is estimated to be between −1 to 0 (Group A minus Group B). After discussing with several experienced statisticians, we finally determined the non-inferiority margin of this study to be −0.5, which is the middle value between −1 and 0.

The evaluation will be conducted at 18-month and 30-month follow-up. Non-inferiority testing will only be performed for the primary endpoint, the number of arrested surfaces after 30 months. Additionally, a Student’s *T* test will be performed to study the effects of the treatment on the number of arrested caries surfaces at the 18-month examination. As different variables might provide different levels of influence on outcome variables, this research will also study the effect modification. The variables that are considered to possibly modify the treatment effects on the outcome variable include the patient’s gender, baseline caries experiences, treatment group assignment, oral hygiene habits, fluoride agent use, parent-assisted tooth-brushing, dental visit behaviour, snacking habits, parental educational level, total family income, and family status (single-parent or two-parent households).

We expect that topical application of 25 % AgNO_3_ solution followed by 5 % NaF varnish and of 38 % SDF solution can both effectively arrest ECC. The results of this study will add to the clinical trial information on the use of AgNO_3_ and NaF and will help to determine whether 25 % AgNO_3_ solution and 5 % NaF varnish are as effective as 38 % SDF solution in arresting childhood caries. Lower concentrations of silver and fluoride are contained in 25 % AgNO_3_ solution and 5 % NaF varnish, respectively, than in 38 % SDF solution; therefore, 25 % AgNO_3_ solution and 5 % NaF varnish are more favourable for use in young children. Because its use for caries management is painless, simple, low-cost, and approved in many countries, 25 % AgNO_3_/5 % NaF could be widely recommended and promoted as an alternative treatment to conventional invasive caries management, particularly among child patients who are too young for conventional dental care.

## Trial status

This randomised clinical trial has been registered in *ClinicalTrials.gov (U.S.)* under the registration number NCT02019160 on 11 December 2013. The recruitment of participating kindergarten children and their parents is in progress from 15 September 2014.

## Additional file

Additional file 1:
**Questionnaire survey.** (DOCX 15 kb)

## Abbreviations

AgNO_3_: silver nitrate
CPI: Community Periodontal Index
dmfs: decayed, missing, filled surfaces score
ECC: early childhood caries
FDI: World Dental Federation
HKU/HAHKW IRB: The Institutional Review Board of the University of Hong Kong/Hospital Authority Hong Kong West Cluster
NaF: sodium fluoride
SDF: silver diamine fluoride
US: United States
VPI: visible plaque index
