# Supplementary material for: The effectiveness of the biannual application of silver nitrate solution followed by sodium fluoride varnish in arresting early childhood caries in preschool children: study protocol for a randomised controlled trial
Source: Trials. 2015 Sep 25;16:426. doi: 10.1186/s13063-015-0960-2 (PMC4582730; doi:10.1186/s13063-015-0960-2)
Supplement: Additional file 1: — Questionnaire survey. (DOCX 15 kb) [file 13063_2015_960_MOESM1_ESM.docx]

**Supporting document: Questionnaire survey**

**Parental Questionnaire**

**Part A: Eating Habit of your child**

1. Is your child **currently bottle-fed** with milk or sugary drinks before sleeping?

□ Yes □ No

2. When does your child **stop bottle-feeding** with milk or sugary drinks?

□ Breast feeding only

□ 1 to 12 months old

□ 13 to 18 months old

□ 19 to 24 months old

□ After 24 months old

□ Still using bottle feeding

□ Others, please specify: __________________

3. Did your child snacked yesterday?

(e.g., soft drinks, juice, snack, candy, biscuits, fruits, desserts)

□ Yes, Please indicate no. of times _______

□ No

**Part B: Oral Hygiene Habits of your child**

4. When did your child start tooth brushing?

□ 1 to 12 months old

□ 13 to 18 months old

□ 19 to 24 months old

□ After 24 months old

□ No tooth brushing yet

5. How many times does your child brush everyday?

□ No/Less than once □ Once　 □ Twice □ More than twice

6. Does anyone assist your child in brushing his/her teeth?

□ Yes　　□ No

7. Does your child use fluoride toothpaste in tooth brushing?

□ Yes　　□ No

□ Not sure, please specify the brand of toothpaste used:______________________

8. Does your child use fluoride mouth-rinse?

□ Yes　　□ No

□ Not sure, please specify the brand of mouth-rinse used:_____________________

**Part C: Dental Visit of your child**

9. Does your child have dental check up? (Not include emergency dental care)

□ Yes □ No

10. Have your child ever seen a dentist for toothache or dental decay?

□ Yes □ No

**Part D: Personal Information of your child**

11. Name of child : _________________

12. Gender : □ M　　□ F

13. Date of birth : ____/____/______ (DD / MM / YYYY)

14. Place of birth : □ Hong Kong 　□ Mainland China 　□ Others, please specify: _________

15. Contact number : ________________ (for follow-ups)

**Part E: Other Information**

16. Home situation: □ Both parents □ Single parent

17. Monthly family income:

□ HK $ 10,000 or below

□ HK $ 10,001 - 20,000

□ HK $ 20,001 - 30,000

□ HK $ 30,001 – 40,000

□ HK $ 40,001 or above

18. Parent education level: Father Mother

| Primary school or below | □ | □ |
| --- | --- | --- |
| Secondary school up to S3  Secondary school complete S5 | □  □ | □  □ |
| College or above | □ | □ |
|  |  |  |

19. Who usually take care of your child? (Please choose one)

□ Parents □ Grandparents □ Domestic helper □Others(e.g., relatives)

**Thank you**
